# Direct formation of gold nanorods on surfaces using polymer-immobilised gold seeds

**DOI:** 10.3762/bjnano.7.72

**Published:** 2016-06-06

**Authors:** Majid K Abyaneh, Pietro Parisse, Loredana Casalis

**Affiliations:** 1 Diamond Light Source Ltd., Harwell Science and Innovation Campus, OX11 0DE Didcot, Oxfordshire, UK; 2 Elettra – Sincrotrone Trieste S.C.p.A., s.s. 14 km 163.5 in Area Science Park, Basovizza, 34149, Trieste, Italy

**Keywords:** atomic force microscopy (AFM), direct surface growth, gold nanorods, nanocomposites, poly(methyl methacrylate) (PMMA)

## Abstract

Herein, we present the formation of gold nanorods (GNRs) on novel gold–poly(methyl methacrylate) (Au–PMMA) nanocomposite substrates with unprecedented growth control through the polymer molecular weight (*M*_w_) and gold-salt-to-polymer weight ratio. For the first time, GNRs have been produced by seed-mediated direct growth on surfaces that were pre-coated with polymer-immobilised gold seeds. A Au–PMMA nanocomposite formed by UV photoreduction has been used as the gold seed. The influence of polymer *M*_w_ and gold concentration on the formation of GNRs has been investigated and discussed. The polymer nanocomposite formed with a lower *M*_w_ PMMA and 20 wt % gold salt provides a suitable medium for growing well-dispersed GNRs. In this sample, the average dimension of produced GNRs is 200 nm in length with aspect ratios up to 10 and a distribution of GNRs to nanoparticles of nearly 22%. Suitable characterization techniques such as AFM and SEM have been used to support concept of the proposed growth method.

## Introduction

Gold nanorods (GNRs) are among the most interesting noble metal one-dimensional (1D) nanostructures and have attracted many researchers and scientists. GNRs exhibit strong tunable plasmonic fields and are biocompatibile, which makes them promising candidates for various applications [1–2]. In many applications, it is necessary to form and distribute 1D nanostructures on a given surface. This can be achieved by either direct surface growth or by an indirect distribution approach. In the latter method, pre-synthesised nanorods (NRs) can form a self-assembled structure by means of chemically modified bonding on surfaces [3–4]. In direct surface growth, formation of NRs or nanowires (NWs) occurs directly on the surfaces using small metal nanoparticles as seeds to grow the NRs, similar to the direct growth of carbon nanotubes and semiconductor 1D nanostructures from catalytic seeds [5]. Direct growth of GNRs on surfaces has been reported in many publications [6–8]. Au seed particles usually bond to the pre-functionalised surfaces using various chemical linkers [9–10]. The substrate is then immersed in a growth solution, which results in the growth of surface-bound seeds into 1D nanostructures, quite similar to seed-mediated growth in solution. Seed-mediated growth is one of the prevailing techniques that have been used in the last years [11]. Controlling the length and aspect ratio of NRs also can be achieved by varying the ratio of seeds to metal salt [12]. GNRs are routinely produced using seed-mediated synthesis techniques [13]. In this growth technique typically a “seed solution” containing small gold nanoparticles is added to a “growth solution” in which particles grow by slow diffusion of gold atoms onto the surface of the seeds to form GNRs. The growth solution contains Au(III) ions, a reducing agent and a surfactant. Many efforts also have been made to align noble metal NRs or NWs with various techniques, such as electron-beam lithography [14–15], Langmuir–Blodgett (L–B) methods [16], stretched polymer matrices [17], self-assembly [18–20], and electric fields [21].

However, both direct and indirect surface-growth approaches present significant challenges. They require complicated protocols to be followed and have to be modified for different surfaces. Developing a technique to facilitate the direct fabrication of 1D nanostructures on any surface with various shapes and sizes is poised to inspire large-scale and mass production of consumer devices. To the best of our knowledge, there have been no reports to date of using gold seeds embedded in an organic polymer for direct surface growth of GNRs. Our work provides the experimental proof-of-principle that GNRs can be grown directly on any surfaces pre-coated with a layer of Au–PMMA nanocomposite formed by UV photoreduction. PMMA has amazing properties such as transparency, flexibility and light weight. Moreover, it is able to immobilise the nanoparticles avoiding their agglomeration and thus maintaining the novel size-dependent properties of nanomaterials. PMMA is widely used in lithography and it is continuously finding new and unique applications in different fields [22]. Combining the exceptional properties of GNRs and PMMA will enable many novel applications to be found in a variety of areas.

There are several reasons why this work is promising and we envisage that fundamental concepts detailed in this report could open up new opportunities for various practical applications such as nanoelectronic, sensing, optoelectronic, and plasmonic devices. Firstly, controlling shape and size of 1D nanostructures is crucial for the investigation of novel properties of these promising materials. Secondly, the synthesis protocol applied in this work can be broadened to produce 1D nanostructures other than GNRs. Thirdly, applying direct growth of NRs on the surface makes this method perfectly suited for many applications such as optical data storage [23], optical laser writing and patterning [24], photocatalysis [25], chemical sensing, biosensing [26–27] and surface-enhanced Raman spectroscopy (SERS) [28]. Last but not least, the synthesis process can be easily extended to screen printing or other thick film deposition processes for batch synthesis procedures [29].

## Results and Discussion

**Figure 1:**
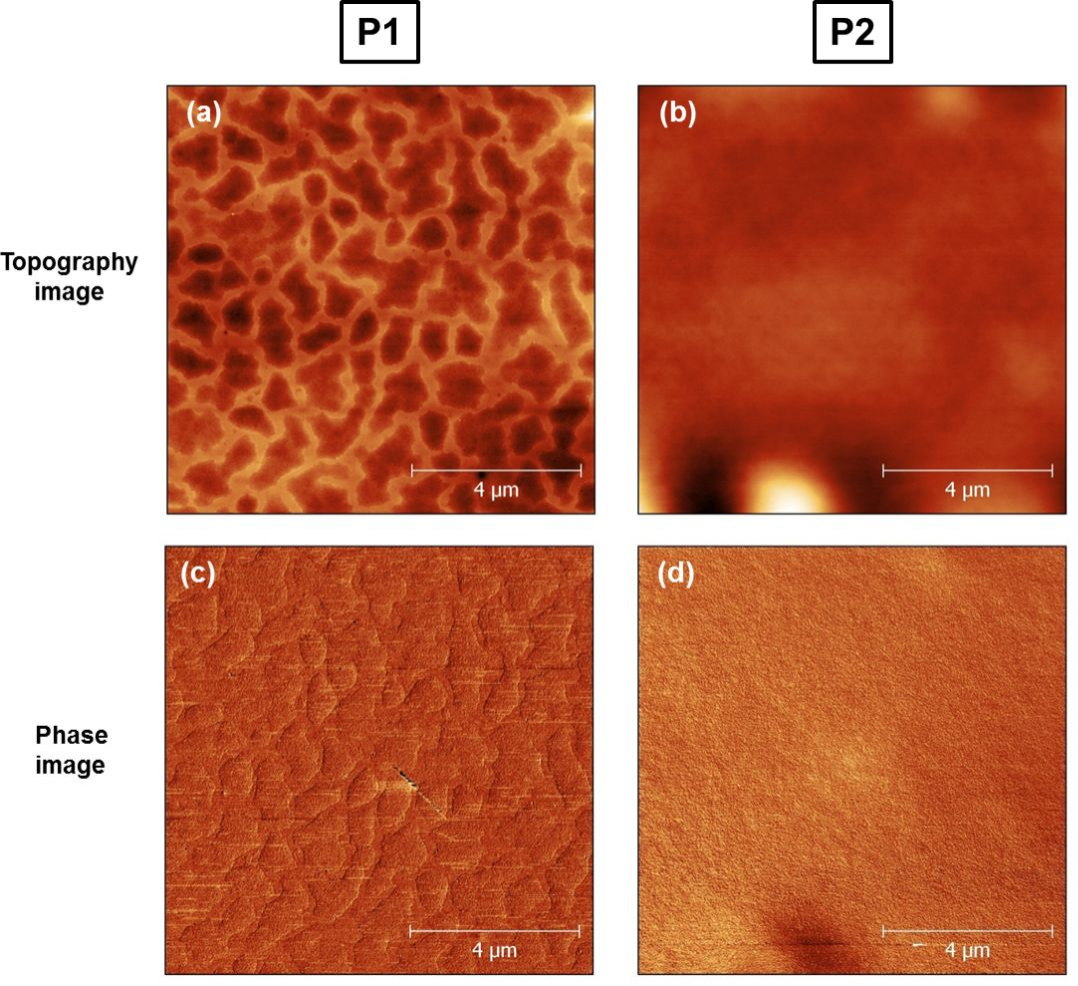
(a,b) AFM topography and (c,d) phase images of UV-irradiated PMMA films on Si wafers for low *M*_w_ (P1) and high *M*_w_ (P2) polymers.

There are numerous experiments and efforts to investigate the formation of gold nanoparticles in a polymer matrix and also to produce well-defined geometries of gold nanoparticles by using photoreduction [30–31]. It has been shown that by using different concentrations of gold salt, one can control the shape as well as the size of the nanoparticles in polymer matrix [30]. The formation of metal–polymer nanocomposites by UV irradiation is a complex process and it was considered to be important to investigate the effect of irradiation time, polymer species as well as the molecular weight of the polymer on the formation of nanoparticles. To explore this, we selected PMMA with two different *M*_w_ and initially investigated the effect of UV irradiation on the neat polymers. Figure 1 shows AFM images of UV-irradiated PMMA films spin-coated on Si wafers for low *M*_w_ (P1) and high *M*_w_ (P2). Figure 1a and Figure 1b are topographic AFM images, and Figure 1c and Figure 1d are AFM phase images for both polymer samples P1 and P2.

The AFM phase images are very helpful for distinguishing different compounds in an AFM image. In fact, while the topographic images show a real three-dimensional mapping of the sample, the phase images are sensitive to variations in the chemical/mechanical properties of the sample [32–33]. Therefore, phase imaging allows for the qualitative assessment of different components of the sample surface. The AFM image of P1 in Figure 1a shows that the exposure of the PMMA to UV light results in a significant roughening of the sample surface. It is believed that these surface modifications lead to important variations of the mechanical properties of PMMA [34], which correlate with the chemical changes resulting from main-chain scission and removal of the ester group [35]. The surface of PMMA with higher *M*_w_, shown in Figure 1b, exhibits a different morphology with micro-roughening and larger swelling domains than P1, caused by UV exposure. It is known that *M*_w_ can influence the PMMA surface morphology after UV irradiation [36]. All these chemical and physical changes in the polymer surface induced by UV irradiation suggest that the role of polymer *M*_w_ in the formation of nanoparticles cannot be ignored.

**Figure 2:**
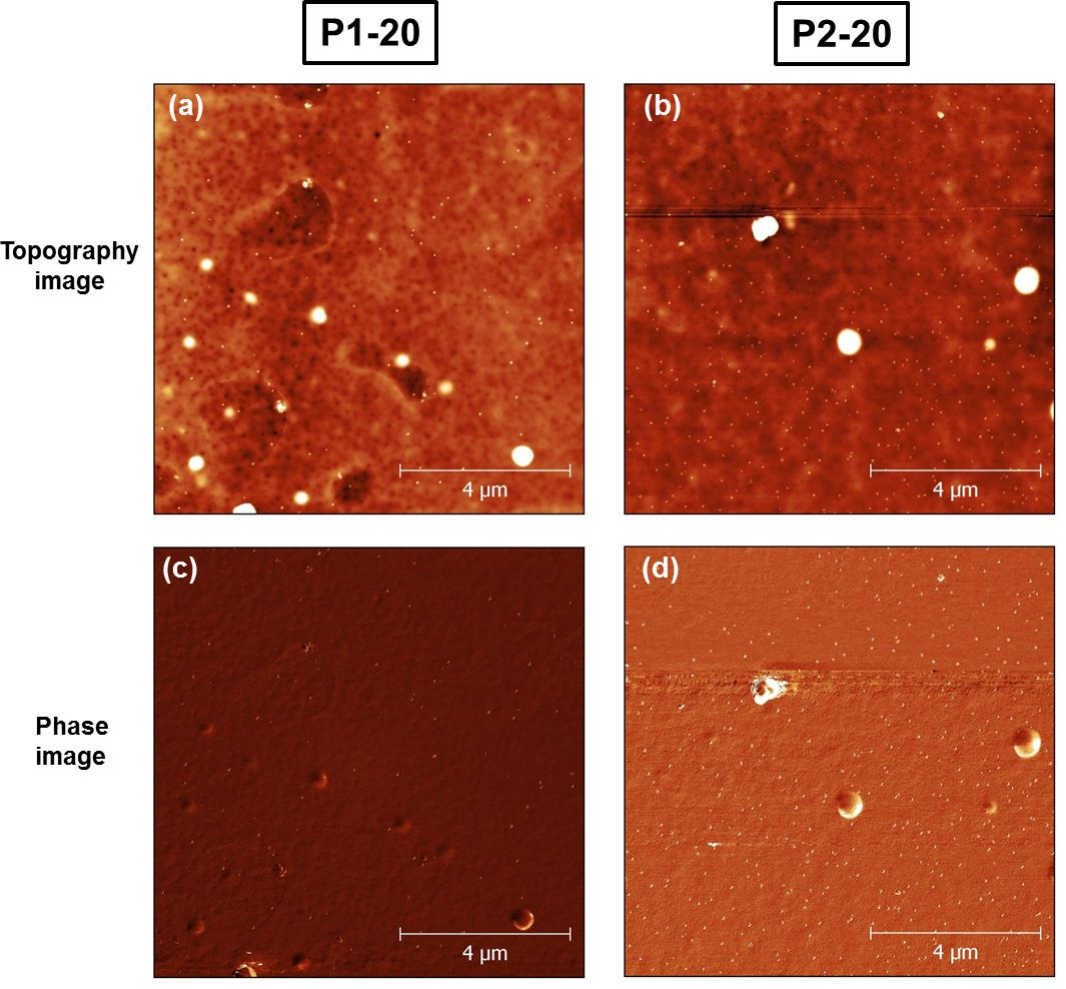
(a,b) AFM topography and (c,d) phase images of 20 wt % Au–PMMA nanocomposites of samples with low *M*_w_ (P1-20) and high *M*_w_ (P2-20).

Hence, we have fabricated Au–PMMA nanocomposites using UV photoreduction for two different selected *M*_w_ of PMMA. The formation of gold nanoparticles in PMMA matrices with low and high *M*_w_ with 20 wt % gold salt is shown in Figure 2. P1-20 denominates the Au–PMMA nanocomposite with low *M*_w_ and P2-20 is for the polymer with high *M*_w_. Figure 2a and Figure 2b show AFM topographical images and Figure 2c and Figure 2d show AFM phase images of P1-20 and P2-20 nanocomposite samples. The images show that gold particles are distributed well at the surface of PMMA with mean particles size of 150 nm for P1-20 and 80 nm for P2-20 nanocomposites. Gwyddion, a multi-platform modular free software [37] for visualization and analysis of data from scanning probe microscopes has been used to process the AFM data and measure the particle size distribution in our samples. The AFM phase image in Figure 2c and Figure 2d are very helpful in identifying the bright spots in Figure 2a and Figure 2b and to establish whether they are big particles or not. The spots appear as the same colour as the surrounding polymer matrix, and formed Au nanoparticles can be observed on top of them. This means that there are few blisters formed on the polymer surface during UV irradiation.

**Figure 3:**
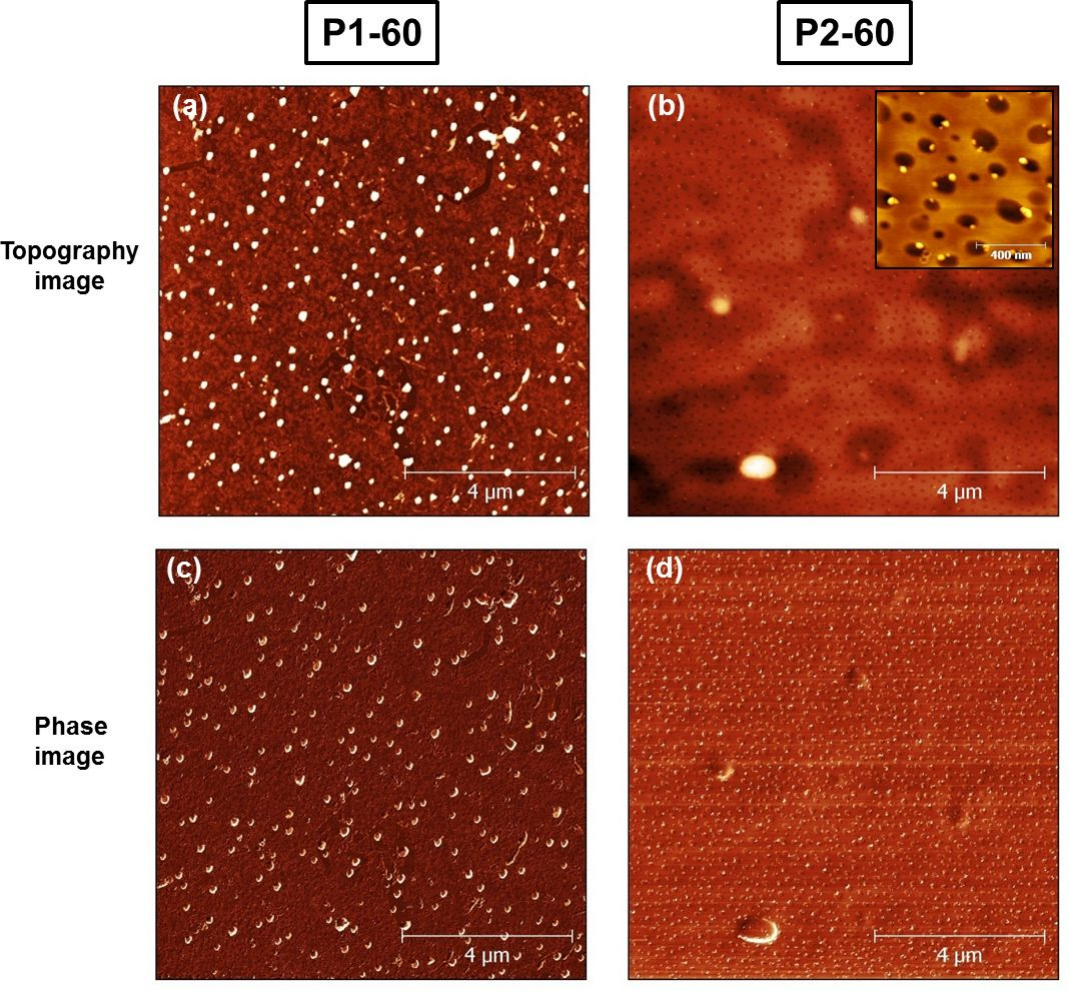
(a,b) AFM topography and (c,d) phase images of 60% Au–PMMA nanocomposites with low *M*_w_ (P1-60) and high *M*_w_ (P2-60). Inset in (b) shows a magnified AFM image for the P2-60.

Figure 3 shows AFM images for low- and high-*M*_w_ PMMA matrices with 60 wt % gold salt which are denominated as P1-60 and P2-60, respectively. The average particles size is 220 nm for P1-60 and 50 nm for P2-60 nanocomposites. The inset in Figure 3b shows a magnified AFM image for P2-60. It can be seen that the formation of the nanoparticles by UV photoreduction is accompanied by the formation of tiny holes and bubbles on the PMMA surface. It is proposed that the combination of UV irradiation and heating can cause tiny holes on the PMMA surface due to vaporizing of products with small *M*_w_ that are generated through light-induced decomposition [38]. In our samples, the association of tiny holes with the gold nanoparticles suggests that the nanoparticles act as local heating sources after their formation in the polymer matrix. This effect can be tuned by decreasing the irradiation dose, or changing irradiation power or exposure time.

**Figure 4:**
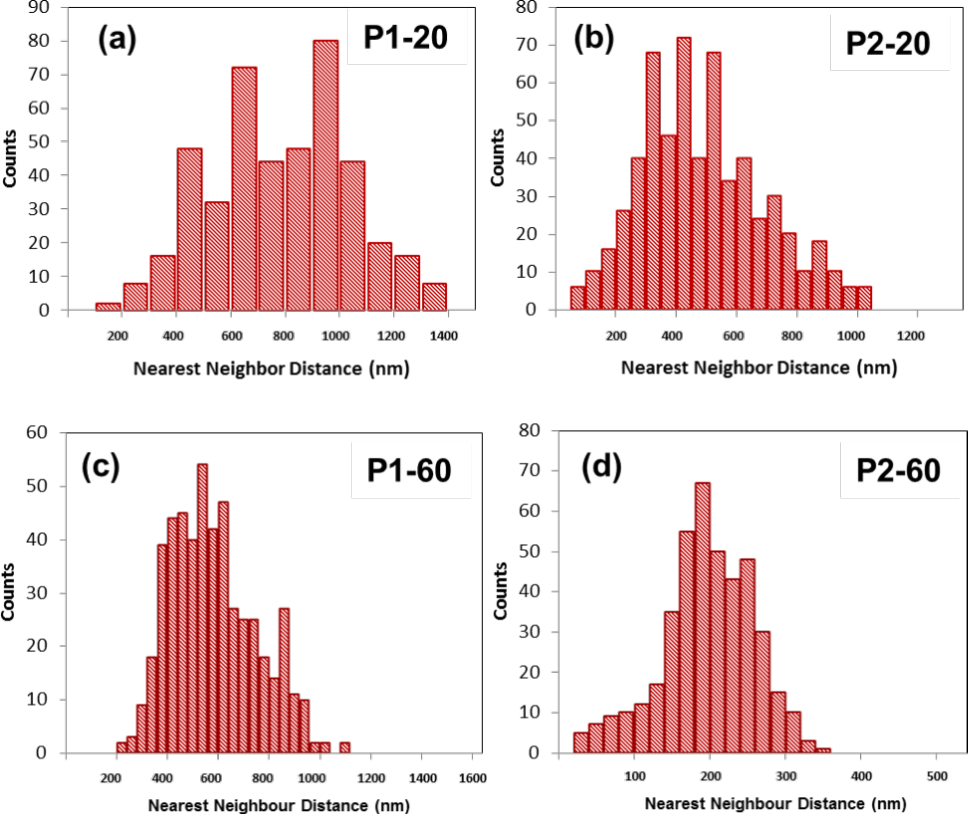
Histogram for the nearest-neighbour distance of gold nanoparticles formed in the (a) P1-20, (b) P2-20, (c) P1-60 and (d) P2-60 nanocomposites by UV irradiation.

Figure 2 and Figure 3 indicate that a broad size distribution of nanoparticles is formed in Au–PMMA nanocomposites and their distance distribution is quite random over the polymer surface. By contrast to the low *M*_w_ polymer, the gold nanoparticles in the high *M*_w_ PMMA matrix are smaller in size and distributed more homogenously on the polymer surface. Figure 4 represents histograms of the nearest-neighbour distance of the gold nanoparticles in the AFM images of Figure 2 and Figure 3. Delaunay triangulation was used for calculating the nearest neighbour distance of the nanoparticles [39]. The average distance between nanoparticles in the P1-20 sample is 800 nm while for P2-20 and P1-60 nanocomposite it is 500 nm with a wide distribution (±500 nm). The average distance for the P2-60 nanocomposite is 180 nm with a narrower distribution (±50 nm).

**Figure 5:**
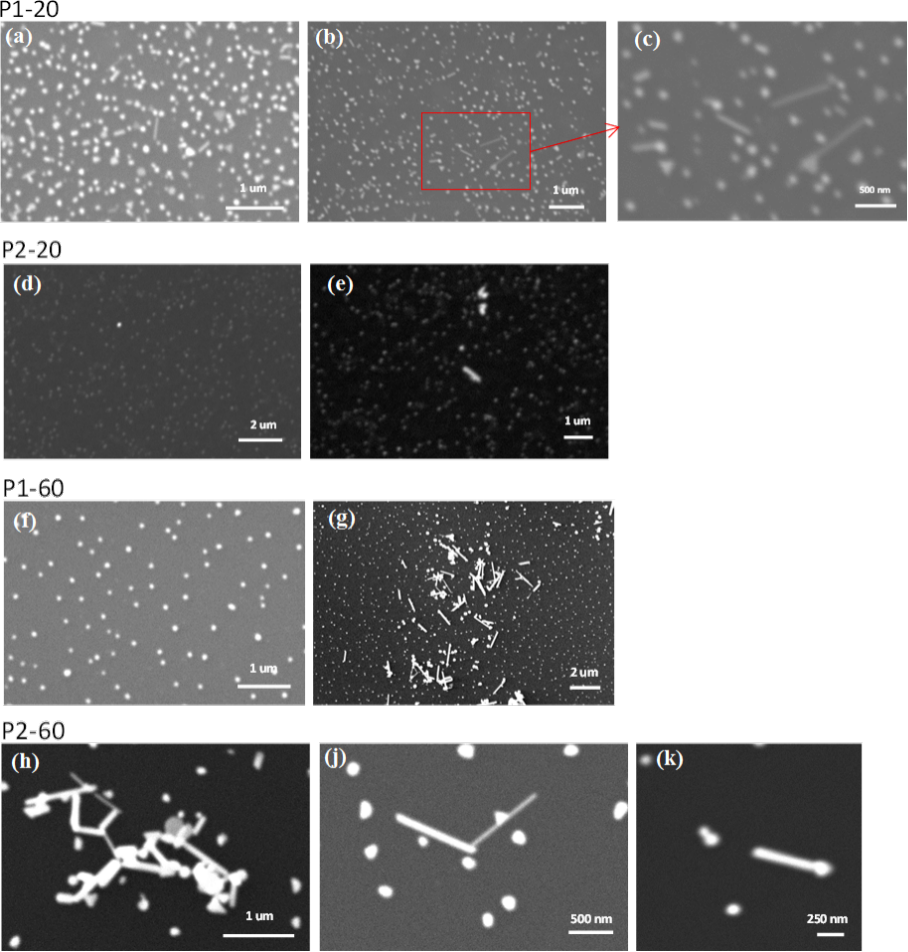
SEM images of different regions of Au–PMMA nanocomposites. (a–c) Low *M*_w_ PMMA with 20 wt % Au salt (P1-20); (d,e) high *M*_w_ PMMA with 20 wt % Au salt (P2-20), (f,g) low *M*_w_ PMMA with 60 wt % Au salt concentration (P1-60) and (h–k) high *M*_w_ PMMA with 60 wt % Au salt concentration (P2-60).

In all samples, the gold nanoparticles are well dispersed in the polymer matrix and no agglomeration is observed. These well-dispersed and protruded gold nanoparticles on the PMMA surfaces offer the possibility of providing a platform and substrate for various applications where individual particles attached to the surface are required. We have shown the potential for using the Au–PMMA nanocomposites as polymer-immobilised Au seeds onto which GNRs can be grown. Figure 5 shows the SEM images of GNRs that are grown on Au–PMMA nanocomposite surfaces by using the seed-mediated direct growth protocol described in the Experimental section. SEM images in Figure 5a and Figure 5b are taken for P1-20 in two different regions and Figure 5c is a magnified image of the red marked region of Figure 5b. GNRs are formed individually over the surface with an average diameter of 50 nm and aspect ratio from 2 to 10 along with triangular shaped nanoparticles. Figure 5d and Figure 5e are showing SEM images taken of the P2-20 sample in distinct regions. Only few GNRs could be observed over a large area of the P2-20 sample and the ratio of GNRs to gold nanoparticles after growth is substantially lower than that of the P1-20 sample. SEM images shown in Figure 5f and Figure 5g are taken of the P1-60 sample in two different regions. As depicted in Figure 5f, the major part of the surface of P1-60 has remained unchanged with dispersed spherical nanoparticles. It is observed that GNRs are formed in a few small regions and agglomerated with larger particles as shown in Figure 5g. Figure 5h, Figure 5j and Figure 5k show how GNRs are formed on the surface of the P2-60 nanocomposite sample. Combinations of individual GNRs and agglomerated rods are observed randomly all over the surface of P2-60. Particles that did not take part in the formation of GNRs have grown into larger particles with different shapes.

These results are a proof-of-concept that UV-photoreduced Au–PMMA nanocomposites provide a suitable seed base for direct growth of GNRs on their surfaces, without any surface modification for chemical bonding agents or linkers. The best distributed GNRs were produced on the P1-20 surface with low *M*_w_ (P1) and 20 wt % of Au salt. This provides an important potential growth control, which can be possibly explained and addressed as follows: It is shown in our previous results [30] that the combination of PMMA with relatively low *M*_w_ and 20 wt % gold salt provide an appropriate medium for the formation of defined morphologies of gold particles with enhanced average crystallite size and preferred growth planes of {111}. On the other hand, it is proposed [40] that cetyltrimethylammonium bromide (CTAB), used as a surfactant in this work, has a preferential binding to specific side faces of gold particles, slowing down the nucleation on these side planes and allowing growth of GNRs on (111) end faces which are dominant in the P1-20 sample.

We believe that this protocol for the growth of GNRs can be further improved. In order to provide better control over the formation of NRs , which in turn will facilitate the tuning of size and aspect ratio of the NRs, further research will be needed to optimize the parameters involved in the process. In addition, it is crucial to study the chemical composition of the GNRs samples using suitable spectroscopic techniques. Hence, full characterization of GNRs samples are also in progress and will be presented in a separate paper.

## Conclusion

For the first time, GNRs have been produced by a novel seed-mediated direct growth route on solid surfaces pre-coated with Au–PMMA nanocomposites formed using UV photoreduction. Protruding gold nanoparticles over the polymer surface provide a well-dispersed seed base for growth of GNRs. The nanocomposite formed with PMMA of lower *M*_w_ and 20 wt % gold (P1-20) provide a suitable medium for growing well-dispersed GNRs. The average dimension of GNRs in sample P1-20 is 200 nm in length with aspect ratios up to 10 and a relation of GNRs to gold nanoparticles after growth of nearly 22%. This demonstrates the potential control over shape and growth distribution of the produced GNRs using polymer-immobilised gold seeds. The underlying concepts represented in this work can be extended to produce other 1D nanostructures.

## Experimental

**Figure 6:**
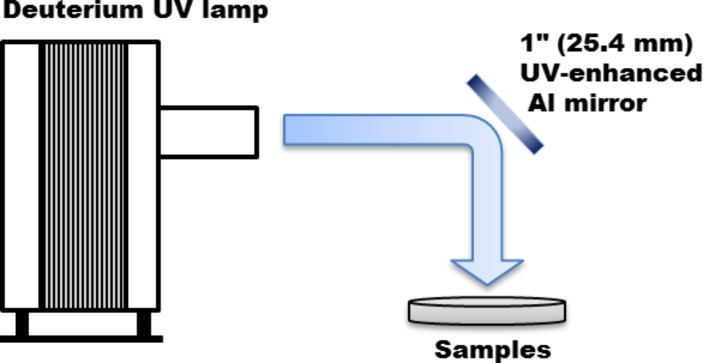
Set-up for UV exposure on the samples using a deuterium UV lamp with diverted beam set-up using an UV-enhanced aluminium flat mirror.

Au–PMMA nanocomposites have been synthesized by using an in situ photo-reduction procedure without using any surfactant, capping agent or reducing agent in the solid form. Fabrication of Au–PMMA nanocomposites using UV irradiation has been reported previously in [30]. Briefly, PMMA with two different molecular weights, *M*_w_, was chosen (P1 with *M*_w_ = 120 kDa, *d* = 1.188 and P2 with *M*_w_ = 996 kDa, *d* = 1.250) and dissolved in acetone to prepare solutions with a concentration of 100 g/L each. Hydrogen tetrachloroaurate(ΙΙΙ)·trihydrate (H[AuCl_4_]·3H_2_0) was then dissolved into the polymer solutions to obtain 10, 20, 40 and 60 wt % gold–polymer samples. Subsequently, the final solutions were deposited on substrates (silicon wafers, glass or Si_3_N_4_ membranes) through spin-coating. Finally, these films were kept under a DC deuterium 30 W UV lamp for 24 h. The UV lamp is operated at 310 mA and 72 V. A UV-enhanced aluminium flat mirror from Thorlabs Inc. was used to divert the UV beam vertically down on the table-top to expose the samples. The setup is shown in Figure 6. These Au–PMMA nanocomposite samples were used as gold-seeds base to grow GNRs on their surfaces. The solid substrates covered with gold seeds embedded in the PMMA matrix were placed into a growth solution containing 5 mL of 0.2 M cetyltrimethylammonium bromide (CTAB), 250 μL of 0.01 M AuCl_4_^−^, 50 μL of 0.1 M ascorbic acid and 20 μL of 0.1 M HNO_3_ and kept undisturbed overnight at room temperature (ca. 24 °C). This step leads to the growth of protruded gold seeds into GNRs via seed-mediated growth.

Atomic force microscopy (AFM) images were recorded using a NTMDT Solver Pro instrument. We have operated it in dynamic mode with silicon cantilevers (NSG30-NTMDT, force constant 40 N/m) which were working at their resonance frequency with an oscillation amplitude in the range of 100–200 nm. Scanning electron microscopy (SEM) images were recorded using a JEOL JSM – 6610LV microscope with 20 kV operating voltage.
